# Reliability and Validity of the Dutch Translation of the Filial Maturity Measure in Informal Caregivers

**DOI:** 10.1007/s10804-015-9207-4

**Published:** 2015-02-24

**Authors:** S. Van Bruggen, C. Bode, P. M. ten Klooster, L. I. M. Lenferink

**Affiliations:** 1 Department of Health Care Ethics and Health Law, Leiden University Medical Center, Leiden, The Netherlands; 2 Department of Psychology, Health and Technology, University of Twente, P.O. Box 217, 7500 AE Enschede, The Netherlands

**Keywords:** Filial maturity, Informal caregivers, Validation, Translation, Filial maturity measure (FMM)

## Abstract

This study explored the reliability and validity of a Dutch translation of the 10-item Filial Maturity Measure (FMM) in a sample of Dutch informal caregivers. The FMM was translated with a forward–backward method and completed by 93 informal caregivers (62 % response rate) with a need dependent parent. Dimensionality of the Dutch FMM was examined by principal component and internal consistency analyses. Criterion validity was examined by assessing correlations with filial love, filial autonomy and level of closeness between parent and child. Construct validity was tested by examining associations with the traits openness and agreeableness. In addition, the relationship with state and trait affectivity was explored. After removal of the item “I worry about turning out like my parent”, the original dimensional structure, internal consistency, criterion and construct validity were confirmed. Additional exploration of the relation between the FMM subscales and trait and state affectivity scales demonstrated that filial maturity is at most weakly associated with trait affectivity. Both FMM scales showed a positive partial correlation with negative state affectivity. The Dutch FMM appears to be a reliable and valid instrument for measuring filial maturity of informal caregivers who provide care to their need dependent parent. The (non-)functioning of one item pointed to the necessity to validate the FMM, but also questionnaires in general in different populations.

## Introduction

Due to increases in life expectancy and the overall aging in Western societies (European Commission 2012), the nature and dynamics of familial relationships have undergone a dramatic shift. This is illustrated by several developments. In the first place, the amount of years that an adult has at least one living parent has increased sharply (Murphy and Grundy 2003). Thus, parent and adult child move together through adulthood and often into old age. To illustrate, in the United States, 13 % of 40 plus adults provide filial care, and another 73 % expect this to be likely in the future (Pew Research Center 2013). In this context, it should also be mentioned that a growing number of 75 plus adults in the United States even moves in with their children (Pew Research Center 2010).

Second, the care adult children eventually provide to their parents has changed. Diseases that used to be fatal have become chronic illnesses (RIVM 2012). As a result, when older adults are care dependent on others, this is for a longer period of their life than in the past, and their care needs have become more complex as well (Brody 1985). When the care needs of the aging parent become complex, the amount of support provided by adult children also increases since many consider providing support as a substantial part of the relation with the aging, care dependent parent (Silverstein et al. 2006).

In order to deal adequately with the care needs of the aging parent, Blenkner (1965) introduces the concept of filial maturity. According to Blenkner, at a certain moment, adult children are confronted with their parent’s frailty and possible need for care and support from the child. As a result, this may lead to a period of filial struggle, in which they have to face the filial task of learning to deal with the changed situation. When they succeed in the accomplishment of this new filial role, they can reach filial maturity.

Developing filial maturity may be relevant for adult children, since the growing care dependence of the aging parent often leads to an asymmetrical transition in the relation with the care providing adult child in several ways (Fischer 1985). First, the adult child perceives a role reversal since the parent becomes dependent on the care of the child (Johnson 2014). This is even more salient when the parent does not recognize it, which sometimes is the case (Wenzel and Poynter 2014). Second, the health problems of the parent may lead to emotional disengagement and distancing of the parent in the relationship and, conversely, increased involvement of the adult child with the relationship. Finally, the relational hierarchy can change: the adult child gradually takes over the decision making process from the parent (Feinberg and Whitlatch 2002). In addition, Brody (1985) emphasizes that being depended on by one’s parent has a different meaning from being depended on by one’s infant or child. In sum, role reversal, changes in the involvement of parent and adult child as well as taking over the decision making role create a change in the relationship between adult children and their aging parents. As a result, it can be very demanding for adult children to provide care to their care dependent parents (Son et al. 2007; Gallagher et al. 2011). To summarize, the difficulties and burdens which adult children can experience in the caregiving process, can be relieved by developing filial maturity.

Blenkner’s concept of filial maturity, which addresses adult children’s challenge to develop a new, filial mature role in the relationship with their care dependent parent, has been elaborated by several others. For example, Marcoen (1995) and Nydegger (1991) emphasized that, in order to provide adequate care to the aging care dependent parent, filial maturity is required. However, Marcoen and Nydegger differ in their conceptualization of filial maturity. That is, Marcoen describes filial maturity as the “dynamic state of continuous, successful coping with the normative task of parent care in middle-aged adult children’ (Marcoen 1995, p. 127). Marcoen proposes filial maturity as consisting of seven components. Four reflect commitment of the child to provide support, and the others are a sense of filial autonomy, reciprocity from the side of the parent as well as collaboration with siblings in the family. This approach emphasizes the commitment of adult children to provide care in relation to the need for care of their aging parents.

In contrast to Marcoen’s view on filial maturity, Nydegger (1991) following the relational concept of Blenkner, emphasizes the way an adult child relates to his aging parent in the context of his whole lifespan. Nydegger considers filial maturity not as a state, but rather as a process already rooted in the child’s adolescence. This process is characterized by two dimensions: distancing and comprehending. During childhood, the child is practically as well as emotionally strongly dependent on the parent. For adolescents and young adults, an important task is to separate from the parents and to develop their own adult identity (Tanner et al. 2009) Although the decrease in childhood closeness with the parent may occur with disagreement or frustrations, and moving out of the parental home can be difficult and painful for both parent and adult child (Kloep and Hendry 2010; Mitchell and Lovegreen 2009; Bouchard 2014), this can also encourage the adult child’s separation process (Seiffge-Krenke 2013; Smetana 2011; Bucx and Van Wei 2008). The process of distancing enables the adult child to reflect on himself in the child role and to develop a realistic, more objective view on the relationship with the parent. The second dimension, comprehension, which may occur when the child enters the adult’s world of work and partner relations (Buhl 2007) or parenting (Bucx et al. 2010), is described by Nydegger (1991) as a ‘gradual deepening’ of comprehending the parent. When the adult child is able to understand the parent’s world and the way life choices and opinions of the parent have been shaped, the final phase of this development is accomplished.

However, the development towards filial maturity may be interfered by different factors. For example, adult children who don’t share their emotions in an open way with their care dependent parent, reported more negative emotions and less satisfaction in the helping relationship (Martini and Busseri 2010). As a result of negative emotions or conflicts between parent and adult child, the distancing process may escalate (Nydegger 1991). That is, an overload of distancing can make it much more difficult for the adult child to understand the parent in his life circumstances. On the other hand, from a developmental perspective it is known that a lack of separation from the parents in adolescence and emerging adulthood may lead to an impaired manifestation of autonomy in adulthood (Koepke and Denissen 2012) and limited confidence in the own problem solving skills (Pizzolato and Hicklen 2011). In addition, over-dependence can obstruct developing an autonomous, realistic view on the parent (Nydegger 1991).

When either the distancing or the comprehending process is not accomplished, specific problems may arise when the parent becomes in need of care. To specify, when the adult child lacks comprehending, he may not recognize the parent’s need for support. On the other hand, when the adult child’s sense of distancing is underdeveloped, this may cause difficulties in several ways. First, since the adult child feels that the care is never sufficient, feelings of guilt, burn out and depression may arise (Gonyea et al. 2008; Springate and Tremont 2014; Madsen and Birkelund 2013; Roach et al. 2013). Second, if the parent becomes physically or mentally unable to maintain the parental role from the past, the adult child may feel anxious and incapable of providing adequate support—which, in turn, may affect the parent’s ability to accept the care (Fowler et al. 2014). Thus, filial maturity requires successful achievement of both distancing and comprehending.

The definitions of filial maturity as formulated by Marcoen and Nydegger suggest that, in order to be able to provide an aging parent with the support that is needed, a child has to be filially mature. An empirical study by Lang and Schütze (2002) confirmed that adult children who are filially ‘autonomous’—understanding the parent’s current life situation and actual wishes—are more prepared for responding adequately to their parent’s socio-emotional and care needs than adult children who tend to be more dependent on their parent. Therefore, in research on parent–child relationships, it may be useful to assess the filial maturity of adult children empirically.

One of the most elaborated questionnaires for measuring filial maturity is the Louvain Filial Maturity Scale (LFMS) (Marcoen 1995), which measures seven dimensions of filial maturity. Although it has an acceptable to good reliability (Marcoen 1995; Stiens et al. 2006), the length of this 81-item questionnaire is often impractical for research purposes. More recently, based on the work of Nydegger (1991), Birditt et al. (2008) constructed the 10-item Filial Maturity Measure (FMM), a questionnaire which measures filial maturity based on the concepts ‘comprehending’ and ‘distancing’. The dimensional structure, internal consistency and construct validity of the scale have been confirmed in a US based population. Their study showed that filial maturity was associated with moderate to low distancing coupled with high comprehending.

The aim of this study was to develop and evaluate a Dutch version of the FMM. After testing the dimensional structure and internal consistency, several forms of validity in relation to other measures will be examined. In the following section, we will discuss our expectations regarding criterion, internal, convergent and divergent construct validity of the Dutch version of the FMM.

1. The validity of the two subscales was first explored by testing the relation between the FMM scales ‘comprehending’ and ‘distancing’. According to the findings of Birditt et al. (2008) and Marcoen (1995), ‘comprehending’ is moderately and adversely related to distancing. Following the criteria of Cohen (1988), correlations higher than .50 are considered as strong, between .30 and .50 as moderate and correlations lower than .30 as weak. Thus, a moderate negative correlation is expected between ‘comprehending’ and ‘distancing’.
2. The most appropriate instrument to test the criterion validity of the FMM is the LFMS. In his conceptualization of filial maturity, Marcoen (1995) distinguishes ‘filial love’ (originating from early childhood attachments) and ‘filial autonomy’. The construct ‘filial love’ is supposed to relate conceptually with comprehending and ‘filial autonomy’ with distancing. Therefore, applying the principle of criterion validity—which means that the FMM should show empirical association with external criteria (Fayers and Machin 2000)—a strong and negative correlation between ‘distancing’ and ‘filial love’ and between ‘comprehending’ and ‘filial autonomy’ is expected. In the same line, we hypothesize moderately positive correlations between ‘filial love’ and ‘comprehending’ and between ‘filial autonomy’ and ‘distancing’.
3. By investing the relation between filial maturity and the concept ‘closeness’ in the affective relationship between parent and child the convergent construct validity is assessed. Too much closeness may be associated with an overload of comprehending and little distance; too little closeness might cause the opposite. This idea is confirmed by Birditt et al. (2008) for both dimensions. Therefore, based on their results, a moderately to highly positive correlation is expected between closeness and the subscale ‘comprehending’. In correspondence with the conceptual explanation, Birditt et al. found moderately negative correlations with ‘distancing’. Therefore, a moderately negative correlation is expected between ‘closeness’ and ‘distancing’.
4. Divergent construct validity is based on the principle that the FMM should show no or only a low correlation with unrelated constructs. Since Perrig-Chiello and Sturzenegger found at most weak relations between the traits ‘openness’ and ‘agreeableness’ on the one hand, and the LFMS-subscales ‘filial help’ and ‘filial helpfulness’ on the other hand (Perrig-Chiello and Sturzenegger 2001), the divergent validity was tested by analyzing the relationship with personality traits. We expect an at most weak correlation for ‘openness’ and ‘agreeableness’ with regard to ‘comprehending’ and ‘distancing’.
5. In addition, the relationship of filial maturity with state and trait affectivity is explored. It may be possible that emotional stability contributes to finding a mature way of relating to the aging parent—or, the other way around, that the process of filial maturity is negatively affected by emotional instability. The trait neuroticism may be interesting to investigate, because it provides an insight in emotional stability (Costa and McCrae 1988). This includes levels of anxiety, hostility and vulnerability. Therefore, the relation with the neuroticism subscale is explored. Furthermore, actual positive or negative emotions may also affect distancing from or understanding of the parent. Therefore, we also explored the association between the FMM and affectivity as a state.

## Materials and Methods

### Participants

Participants were approached between May 2011 and January 2012. Adult children could participate in our study if they provided unpaid care to a parent for either a minimum of 8 h a week, or since at least 3 months. Participation was anonymous. The integrity of human or animal rights was warranted.

To create a broad sample, participants were approached in several ways. In the western part of the Netherlands, the survey was sent to 72 child caregivers of frail older adults participating in another research project from the Leyden University Medical Center. After 3 weeks, a reminder was sent to the non-responders. Four respondents (6 %) did not respond because their parent recently passed away. The questionnaire was sent back by 52 of the remaining respondents (72 %). Since eight respondents (11 %) did not fit the inclusion criteria and one respondent had not filled in the FMM items (1 %), 43 respondents (60 %) were left for further analysis. In the eastern part of the Netherlands, 77 participants were approached through the snowball method (Atkinson and Flint 2001) and by recruitment in a pharmacy and meetings for informal caregivers of patients with Alzheimer’s disease. From the distributed questionnaires, 54 (70 %) were sent back, four respondents (5 %) were excluded because they did not fit the caregiver criterion. Since all respondents filled in the FMM items, 50 questionnaires were left for further analysis.

In sum, the final dataset consisted of 93 respondents, ranging in age from 24 to 70 years (M = 53.6, SD = 9.3; 66 women, 25 men, 2 missing). The age of the related care receiver ranged from 50 to 101 years (M = 83.1, SD = 9.4; 57 women, 33 men and 3 missing). The amount of support provided per week varied between 1 and 168 h (M = 11.6, SD = 20.4). Table 1 shows the educational background of the respondents, the reason for taking care of their parent and the type of support being given.

**Table 1:** Education level and type of support given by the respondents (n = 93)

Characteristic	N	%
Level of education child		
Primary school	4	5
Secondary school, ground level	16	18
Secondary school, advanced level	18	20
Vocational school, basic level	26	30
Vocational school, advanced level	23	26
University	1	1
*Missing*	*5*	
Reason for informal care		
(Beginning) dementia, cognitive decline	40	43
Psychological problems	10	11
Physical handicaps	41	44
Other serious disease	12	13
Other	29	31
Type of support		
Emotional support	87	96
Accompanying/transport to social events, GP etc.	84	90
Domestic help	81	87
Financial/administrative help	70	75
Personal care	34	37

### Measures

Among the measures which were used to test the validity of the FMM, two were related to the relationship between the adult child and the aging parent. In order to avoid confusion and to ensure that the respondents would answer the questionnaires consistently concentrating on one parent, they were asked to choose the parent to who they provided most care, when filling in the questionnaire.

#### Filial Maturity Measure

The FMM consists of 10 statements about the relation with a parent. The first six items of the FMM are based on the concept ‘comprehending’ (e.g., “It means a lot to me when my parent confides in me”) and item 6–10 on ‘distancing’ (e.g., “My parent has some really annoying habits”). Item 8 (“My parent is practically perfect”) is reversely scored. On a 5 point Likert scale, the respondent can express his agreement with each item (1 = “I strongly disagree”, 5 = “I strongly agree”). A high score on ‘distancing’ or ‘comprehending’ signifies much distancing or understanding.

Through a common backward–forward translation procedure as described by Bracken and Barona (Bracken and Barona 1991), the translation of the FMM into Dutch was realized. Three bilingual English-Dutch language experts were independently involved with the translation process. The first expert translated the scale from English to Dutch. The result was compared with the original formulation. The second expert retranslated the scale blindly—without prior knowledge of the scale—back to English. Since the retranslation of several items was not identical to the original formulation, the procedure was repeated with a third independent expert. Consensus was found on all items, except item 9 (“I worry about turning out like my parent”). It seemed impossible to find perfect agreement on this item. The final formulation of this item was the result of a compromise. An overview of the translated items is presented in “Appendix”.

In an explorative study (Birditt et al. 2008), the internal consistency of the original version of the FMM seemed good (Cronbach’s α = .75 for both ‘comprehending’ and ‘distancing’). In the same study, confirmative analysis proved the stability of ‘comprehending’ (α = .75) and ‘distancing’ (α = .71). In our study, only two respondents had a missing value on an FMM item. In order to avoid biased results, the missing scores have been imputed with the sample means of these items.

#### Other Measures

The validation procedure was carried out with four different established instruments. Both filial love and filial autonomy were assessed with identically labeled subscales of the LFMS, developed by Marcoen (1995). The ‘filial love’ subscale consists of 20 items (e.g., “The relationship with my parent gives support in my life”) and the subscale ‘filial autonomy’ (e.g., “My parent has his/her life, I have my life”) counts 15 items. The internal consistency of both ‘filial love’ and ‘filial autonomy’ (respectively α = .87 and α = .62) in this study corresponded with the values reported by Marcoen (1995). The LFMS has separate editions for mother and father. The formulation of the items is identical, but adapted to the gender of the parent. We replaced the word ‘father’ or ‘mother’ by ‘parent’, so that our survey would be useable for any adult child, regardless of the gender of the parent. Agreement with the statements was measured with a 5 point Likert scale (1 = “I strongly disagree”, 5 = “I strongly agree”). High scores indicated high levels of filial love and autonomy.

The level of closeness between adult child and parent was, following the example of Birditt et al. (2008), assessed with the Inclusion of the Other in the Self Scale (IOS Scale) (Aron et al. 1992). The 1-item IOS Scale is a Venn diagram measuring the closeness in a relationship. The diagram contains seven pictograms which represent the relation between ‘self’ and ‘other’—for our research purpose adjusted to ‘child’ and ‘parent’—varying from distant to symbiotic. The respondent chooses the pictogram that best fits his view of the relational closeness. The IOS scale has been validated against a range of other closeness scales, which showed a modest to high correlation with closeness, intimacy and positive emotions about the other. A high level of symbiosis represents a high closeness as rated by the adult child.

The relation between the FMM and personality traits was analyzed with the Dutch translation (Hoekstra et al. 2003) of three subscales of the NEO Five Factor Inventory (NEO-FFI) (Costa and McCrae 1989), which is a shortened version of the NEO Personality Inventory (NEO-PI) (Costa and McCrae 1985). The subscales ‘openness’ (e.g., “I love playing with theories or abstract ideas”; α = .76), ‘agreeableness’ (e.g., “In general, I prefer working together with others instead of competing with them”; α = .67) and ‘neuroticism’ (e.g., “When I’m under a great deal of stress, sometimes I feel like I’m going to pieces”; α = .84) all consist of 12 statements with a 5 point Likert scale (1 = “I strongly disagree”, 5 = “I strongly agree”). A high score on a subscale corresponds with a high level of openness, agreeableness or neuroticism. In this study sample, the internal consistency of both ‘openness’ (α = .76) and ‘agreeableness’ (α = .67) turned out to be relatively low compared to the original NEO-PI report (α = .89 respectively α = .76, Costa and McCrae 1988).

Positive and negative affect were measured with the Dutch translation (Van Emmerik and Jawahar 2006) of the Positive and Negative Affect Scale (PANAS) (Watson and Clark 1994). The PANAS has two subscales: positive and negative affect. Both subscales consist of ten items which represent positive (e.g., “Excited”; “Strong”; “Enthusiastic”; α = .77) respectively negative (e.g., “Sad”; “Upset”; “Hostile”; α = .87) affects, with a 5 point Likert scale which measures frequency in the last month (1 = “Never”, 5 = “Very often”). A high score on positive or negative affect indicates a high level of positive respectively negative affect. In this study sample, the internal consistency of positive affect was somewhat lower than reported by Watson and Clark (1994) and Van Emmerik and Jawahar (2006) but still satisfying; the internal consistency of the negative affect was similar to their findings.

The survey additionally included items that register a variety of specific caring tasks (five items, e.g.: “Do you provide your parent with personal care?”, “Do you assist your parent with domestic tasks?”, answering options: yes, no), burden (17 items, e.g., “Did you perform your work or other activities with less accuracy than you used to do, since you were so busy with providing care?”, “Did you often lack time in the period that you provided the informal care?”, answering options: yes, not that much, no), motives for caring (14 statements, e.g., “No one else is available”, “The care recipient prefers to be helped by me”). Agreement of respondent with the statement was measured with a 3-level answer scale: of high importance, of considerable importance, of no importance). These items were selected from a national survey, which is conducted on a regular base by the Netherlands Institute for Social Research (SCP). With an open-end question (“On average, how many hours a week do you provide care?”) the participant was asked to indicate the amount of informal care he provided to his parent. These items were used to provide insight in the characteristics of our sample.

## Results

The first aim of this study was to investigate whether the Dutch translation of the FMM had the same factor structure and reliability as the original US version. The first step was to analyze the dimensional structure of FMM with a principal component factor analysis (PCA) with Varimax rotation. PCA is a widely accepted procedure for exploring how test items load on different theoretical dimensions (Bryant 2000; Cicerelli 1988, in Birditt et al. 2008). Although the proportion of explained variance was considerably high (61 %), the items showed an uninterpretable distribution on 3 dimensions. To understand these results, we tested the reliability of the ‘comprehending’ and ‘distancing’ items separately. The reliability of ‘comprehending’ turned out to be good (Cronbach’s α = .76). However, the internal consistency of the 4 ‘distancing’ items was not satisfactory (Cronbach’s α = .60). When zooming in on the influence of each separate item, removal of item 9 (“I worry about turning out like my parent”) led to a considerable increase of Cronbach’s α up to .70 (see Table 2). Thus, the internal consistency of both subscales was warranted.

**Table 2:** Internal consistency of subscale ‘Distancing’ (n = 93)

Subscale ‘Distancing’ items	Cronbach’s α if item deleted
7. Regardless of how much I love my parent, he/she certainly has faults	.470
8. My parent is practically perfect (REVERSED)	.489
9. I worry about turning out like my parent	.697
10. My parent has some really annoying habits	.404

The effect of removing item 9 on the reliability of the ‘distancing’ subscale was reason for repeating the PCA without item 9. In this step, the theoretical constructs ‘comprehending’ (R^2^ = 30 %) and ‘distancing’ (R^2^ = 25 %) were reflected clearly in the factor solution (see Table 3). Even though the distinction between the loading on ‘comprehending’ and ‘distancing’ for item 3 and 6 was not clear cut, the dimensional solution of all items together confirmed both the theoretical framework and the dimensional structure of the FMM.

**Table 3:** Principal component analysis of the 9 item Filial Maturity Scale (n = 93)

	Component	
	Comprehending	Distancing
4. I share my deepest thoughts and feelings with my parent	0.761	−0.292
1. I often tell my parent about my problems and rely on him/her for advice	0.737	−0.319
2. It means a lot to me when my parent confides in me	0.680	0.038
5. My parent sometimes comes to me for advice on important matters	0.616	0.199
3. I think of my parent as more of a friend than a parent	0.580	−0.409
6. As I grow older. I notice my parent and I have more in common	0.523	−0.305
7. Regardless of how much I love my parent, he/she certainly has faults	0.153	.829
10. My parent has some really annoying habits	−0.259	0.777
8. My parent is practically perfect (REVERSED)	−0.199	0.658

As Table 4 shows, a considerable negative correlation (*r* = −.34, *p* < .001) was found between ‘comprehending’ and ‘distancing’, which was in accordance with the findings of both Birditt et al. (2008), Marcoen (1995) and our hypothesis.

**Table 4:** Descriptives, rho correlations and partial correlations of filial maturity scales, IOS scale, NEO-FFI and PANAS. All ρ coefficients are tested on two-tailed level (n = 93)

	Mean	SD	Comprehending		Distancing	
			Spearman coefficient	Partial coefficient	Spearman coefficient	Partial coefficient
FMM comprehending	2.92	0.69			−0.34***	
FMM distancing	3.51	0.78				
LFMS filial love	3.49	0.49	0.61***	0.54***	−0.51***	−0.41***
LFMS filial autonomy	3.20	0.41	−0.34***	−0.28**	0.24*	0.14
IOS scale	4.32	1.86	0.61***	0.55***	−0.36***	−0.21
NEO-FFI openness	3.08	0.54	−0.03	0.03	0.17	0.18
NEO-FFI agreeableness	3.77	0.35	−0.004	−0.05	−0.12	−0.13
NEO-FFI neuroticism	2.43	0.62	0.13	0.20	0.16	0.21*
PANAS positive affect	3.68	0.40	0.13	0.13	−0.03	0.01
PANAS negative affect	2.28	0.55	0.15	0.27**	0.28**	0.36**

* *p* < .05; ** *p* < .01; *** *p* < .001

The next step was to assess the criterion and external construct validity of the FMM. When testing the distributional properties of the scales, the IOS Scale, the NEO-FFI subscale ‘neuroticism’ as well as the PANAS scores appeared not to be normally distributed. To ensure maximal reliability of the results, the relations between both FMM subscales and the validation instruments were analyzed with non-parametrical Spearman coefficients. In addition, in order to control for the moderate association with the other FMM subscale, also partial correlation coefficients were computed.

The majority of the expectations with regard to the relation between the FMM subscales and the LFMS subscales ‘filial love’ and ‘filial autonomy’ was confirmed. ‘Comprehending’ showed a strong positive correlation with ‘filial love’ (*r* = .61, *p* < .001) and a moderate negative correlation with ‘filial autonomy’ (*r* = −.34, *p* < .001). The ‘distancing’ subscale showed the expected opposite pattern: it correlated strongly and adversely with ‘filial love’ (*r* = −.51, *p* < .001). The positive correlation with ‘filial autonomy’ was weaker than expected (*r* = .24, *p* < .05) and, controlling for ‘comprehending’, not significant. However, the main partial-correlational pattern between the FMM and LFMS subscales was similar to the Spearman coefficients.

The expectations with regard to the convergent construct validity were mostly confirmed by the strong positive correlation of the IOS Scale with ‘comprehending’ (*r* = .61, *p* < .001) and the negative and moderate correlation with ‘distancing’ (*r* = −.36, *p* < .001). Even though no significant partial correlation between ‘distancing’ and the IOS scale was found, it was, corresponding with our expectation, still a negative association (*r* = −.21). Our expectations regarding divergent validity were confirmed by the absence of significant Spearman and partial correlations between the FMM subscales and ‘openness’ and ‘distancing’.

Besides testing the validity of the FMM, another aim was to explore whether the concept of filial maturity is related to state or trait affectivity. The absence of a significant correlation between neuroticism and the filial maturity subscales, except a weak significant partial correlation with ‘distancing’ (*r* = .21, *p* < .05), demonstrated that emotional instability did not affect comprehending the parent or taking distance. The PANAS subscale ‘positive affect’ did not show a significant correlation with the filial maturity scales. Although the PANAS subscale ‘negative affect’ had no significant correlation with ‘comprehending’, controlling for ‘comprehending’, a significant partial positive correlation was found (r = .27, *p* < .01). In addition, when controlling for ‘comprehending’, the weak positive correlation between ‘negative affect’ and ‘distancing’ (*r* = .28, *p* < .01) increased (*r* = .36, *p* < .01).

## Discussion

The aim of this study was to assess whether the Dutch translation of the FMM had similar psychometric properties as the American version. The reliability, internal consistency and external validity of our translation are examined and largely confirmed. To begin with, the internal consistency of the FMM will be discussed.

The reliability of the subscale ‘comprehending’ was good. To obtain an acceptable internal consistency of the subscale ‘distancing’ removal of item 9 *(“*I worry about turning out like my parent*”)* was necessary. The poor functioning of item 9 could be explained in several ways. First, in correspondence with the study of Birditt et al. (2008), this item had a lower loading on the factor ‘distancing’ compared to the other items. Second, no unequivocal agreement could be found on this item during the translation process. Apparently, item 9 was hard to grasp or could be understood in more than one way. As a consequence, this ambiguity may also have influenced interpretation by the respondents. Third, the population of this sample differed from the study of Birditt et al. (2008) in age of participants (mean age 53.6 vs. 20.7 years) and sample selection. More specifically, in the study of Birditt et al. participants were recruited via convenience sampling in US recruitment sites, such as college courses at a large university and community festivals without any reference to characteristics of their parents. In contrast, for our study sample, informal caregivers with physically or cognitively need dependent parents were recruited. As a result, our participants may have had a different perception of ‘turning out like my parent’ than people in their twenties and thirties with parents in middle adulthood. To summarize, various factors may have contributed to the unexpected (dis)functioning of item 9. Since removal of item 9 led to an acceptable reliability of the subscale ‘distancing’, a well-fitting factor solution and good internal construct validity, the internal consistency of the FMM may be considered as solid. Our results suggest that the construct filial maturity and the measurement instruments might be sensitive for sample specific characteristics. Therefore, to make sure that the instrument is usable across various groups, the content validity should be tested in various contexts and age groups.

Since the Spearman and partial correlations between the FMM and the LFMS subscales, except the weak relation between ‘distancing’ and the LFMS subscale ‘filial autonomy’, met the majority of our expectations, the criterion validity of the FFM seems acceptable. Moreover, the pattern of associations between the FMM and the IOS Scale as well as the lack of correlation with the NEO-FFI subscales ‘openness’ and ‘agreeableness’ confirmed most of the expectations regarding the convergent and divergent validity of the FMM.

In addition to expected associations of the FMM with related constructs, we also explored the relationship between filial maturity and state or trait affectivity. The at most weak correlations between the FMM subscales and the NEO-FFI subscale ‘neuroticism’ suggest that filial maturity is not related to emotional instability. The same findings were found for positive affect, which did not correlate significantly with the FMM subscales. However, when controlling for the other FMM subscale, negative affect was positively associated with both ‘comprehending’ and ‘distancing’. Since this finding is difficult to explain from the existing knowledge on filial maturity, we recommend further examination of this theme.

To summarize, our findings suggest that filial maturity is a stable concept, which may be viewed as different from a positive state or a lack of emotional stability. This finding might be beneficial from the perspective of coping with the need dependent older parents. Apparently, the ability to respond in an adequate way to the—possibly confrontational—need dependence of the parent is not associated with emotional instability. In other words, if the adult child understands the true needs of the aging parent and, at the same time, is able to maintain a healthy or ‘mature’ distance, he might be better capable to provide the parent with adequate support—even if the adult child lacks emotional stability.

Because the selection of the validation instruments is based on the work of Birditt et al. (2008), the fact that our study confirms their results adds to the consistency of the findings. However, several other interesting issues remain to be studied. For instance, Birditt et al. (2008) already explored which balance between comprehending and distancing would reflect filial maturity. Their study showed that filial maturity of adult children was associated with moderate-to-low distancing coupled with high comprehending. However, in order to “diagnose” a respondent’s degree of filial maturity, it would be useful to further understand how individual scores could be interpreted. In addition, little is known on how filial maturity develops over time. It would be worth zooming in on different stages of adulthood to learn which characteristics of adult children and the parents, such as parental maturity (Mendonça and Fontaine 2014; Pitzer et al. 2011), may influence the rise of a balance between distancing and comprehending, especially since this may create a new sense of closeness which can only be realized in this mature stage of life (Fingerman (2001). A related question is whether informal middle aged caregivers differ in their level of filial maturity in comparison to other groups, such as young adults. To add, on a more fundamental level, more insight in the relationship between the FMM subscales and care related variables is needed. To specify, exploring the relationship between ‘comprehending’ and ‘distancing’ and, say, the capacity to make independent emotional or financial decisions would enrich our understanding of the filial maturity concept in the context of caregiving. Finally, it would be interesting to test whether (a lack of) filial maturity could predict other problems in the relationship between the adult child and the older parent, such as burden of the child when the parent becomes care dependent. Moreover, when tensions in the relationship between the adult child and the parent arise, a better understanding of filial maturity may contribute to providing the child caregiver with adequate support.

To conclude, the Dutch translation of the FMM seems a reliable and valid instrument to assess filial maturity of informal caregivers. To develop an interpretation guide of individual scores and to enhance our understanding of filial maturity in relation to other relevant factors, further research is warranted.

## Appendix

See Table 5.

**Table 5:** Dutch translation of the filial maturity measure

	Original	Dutch translation
1	I often tell my parent about my problems and rely on him/her for advice	Ik vertel mijn vader/moeder vaak over mijn problemen, en vertrouw op op hem/haar voor advies
2	It means a lot to me when my parent confides in me	Het betekent veel voor mij wanneer mijn vader/moeder vertrouwen in mij heeft
3	I think of my parent as more of a friend than a parent	Ik beschouw mijn vader/moeder meer als vriend(in) dan als ouder
4	I share my deepest thoughts and feelings with my parent	Ik deel mijn diepste gedachten en gevoelens met mijn vader/moeder
5	My parent sometimes comes to me for advice about important matters	Mijn vader/moeder komt soms naar me toe voor advies over belangrijke zaken
6	As I grow older, I notice my parent and I have more in common	Naarmate ik ouder word, valt me op dat mijn vader/moeder en ik meer gemeen hebben
7	Regardless of how much I love my parent, he/she certainly has faults	Ongeacht hoe veel ik van mijn vader/moeder houd, hij/zij heeft fouten
8	My parent is practically perfect (REVERSED)	Mijn vader/moeder is zo goed als perfect
9	I worry about turning out like my parent	Ik ben bezorgd dat ik uiteindelijk zo word als mijn vader/moeder
10	My parent has some really annoying habits	Mijn vader/moeder heeft een paar echt vervelende gewoonten
